# Impact of Marital Status on Prognosis of Patients With Invasive Breast Cancer: A Population-Based Study Using SEER Database

**DOI:** 10.3389/fonc.2022.913929

**Published:** 2022-07-22

**Authors:** Dechuang Jiao, Youzhao Ma, Jiujun Zhu, Hao Dai, Yue Yang, Yajie Zhao, Xuhui Guo, Zhenzhen Liu

**Affiliations:** Department of Breast Disease, Henan Breast Cancer Center, Affiliated Cancer Hospital of Zhengzhou University & Henan Cancer Hospital, Zhengzhou, China

**Keywords:** breast cancer, marital status, prognosis, surveillance, epidemiology and end results (SEER)

## Abstract

**Objective:**

This study aimed to investigate the prognostic roles of marital status in patients with invasive breast cancer. Method: We extracted the data of patients with invasive breast cancer who were diagnosed during 2010–2015 and had complete staging and molecular typing from the Surveillance, Epidemiology, and End Results (SEER)-18 database. Kaplan–Meier curve method and Cox regression analysis were performed to investigate the differences in breast cancer–specific survival (BCSS) and overall survival (OS) in the total population and various subgroups with different marital statuses.

**Results:**

Among the 324,062 patients with breast cancer in this study, 55.0%, 40.0%, and 5.0% were married, unmarried, and unknown, respectively; 51.8%, 32.2%, 10.5%, and 5.5% were patients with Stages I, II, III, and IV breast cancer, respectively. The 5-year BCSS and OS of married patients were 92.6% and 88.1%, respectively, higher than those of unmarried patients (88.3% and 78.1%, *P* < 0.001). After adjustment for sex, age, T and N stages, histological grade, insurance status, race, year of diagnosis, and molecular subtypes, married status was an independent predictor of better BCSS [hazard ratio (HR) = 0.775, 95% confidence interval (CI) = 0.753–0.797, *P* < 0.001) and OS (HR = 0.667, 95% CI = 0.653–0.681, *P* < 0.001). After multivariate analysis of various subgroups of sex, age, stage, histological grade, insurance status, race, and molecular subtype, married status was an independent predictor of better BCSS in all subgroups except for Grade IV, age < 35 years, and uninsured subgroups. Marital status was an independent predictor of better OS in all subgroups except the subgroup with age <35 years.

**Conclusions:**

In conclusion, marital status was an independent prognostic factor for breast cancer. The unmarried patients with breast cancer had a worse prognosis, except for the subgroup with age <35 years. Hence, unmarried patients with breast cancer and age ≥35 years may need additional psychosocial and emotional support to achieve more prolonged survival, besides active treatment of primary disease.

## Introduction

Psychosocial factors are closely related to the occurrence and prognosis of malignant tumors, while marital status is one of the most critical psychosocial factors affecting the occurrence and development of malignant tumors (1, 2). Previous studies revealed that the married population had a healthier lifestyle, including a healthy diet, physical exercise, and regular physical examination, which might be intermediate factors in cancer prevention (3). Marital status is closely related to the prognosis of multiple malignant tumors (4–8). Married patients may receive more emotional and financial support, get more standardized and complete medical treatment, and obtain a better prognosis (9–11). In 2020, only 50% of American residents were married, which was a decrease of 9% in the last 25 years. Moreover, this downward trend has always existed. Hence, the relationship between marital status and cancer prevention and treatment is worthy of further research.

As a systematic disease, breast cancer has been considered one of the most affected cancers by marital status. The pain caused by widowhood or divorce and a series of following unhealthy lifestyles are associated with the onset of breast cancer (3, 12). Meanwhile, the lack of experience of pregnancy and lactation in unmarried women may also be related to breast cancer (13–16). Previous studies indicated that unmarried patients with breast cancer were usually diagnosed at an advanced stage and had more depressive symptoms. Additionally, compared with unmarried patients, married patients received more reasonable and standardized treatment (4).

Chen et al. (8) reported that marital status affected the prognosis of patients with breast cancer by affecting the stage at the time of diagnosis. However, the prognosis of breast cancer is affected by various factors. Indeed, other clinical and social indicators, including age, race, insurance status, and sex, are closely related to the marital status and the prognosis of breast cancer. Whether and how these factors affect the relationship between marital status and prognosis remain elusive. Additionally, the molecular typing and patients with the first diagnosis of Stage IV breast cancer were not included in previous studies on the relationship between marital status and prognosis. This study investigated the relationship between marital status and prognosis in patients with different molecular typing and stages.

## Materials and Methods

**Figure 1:**
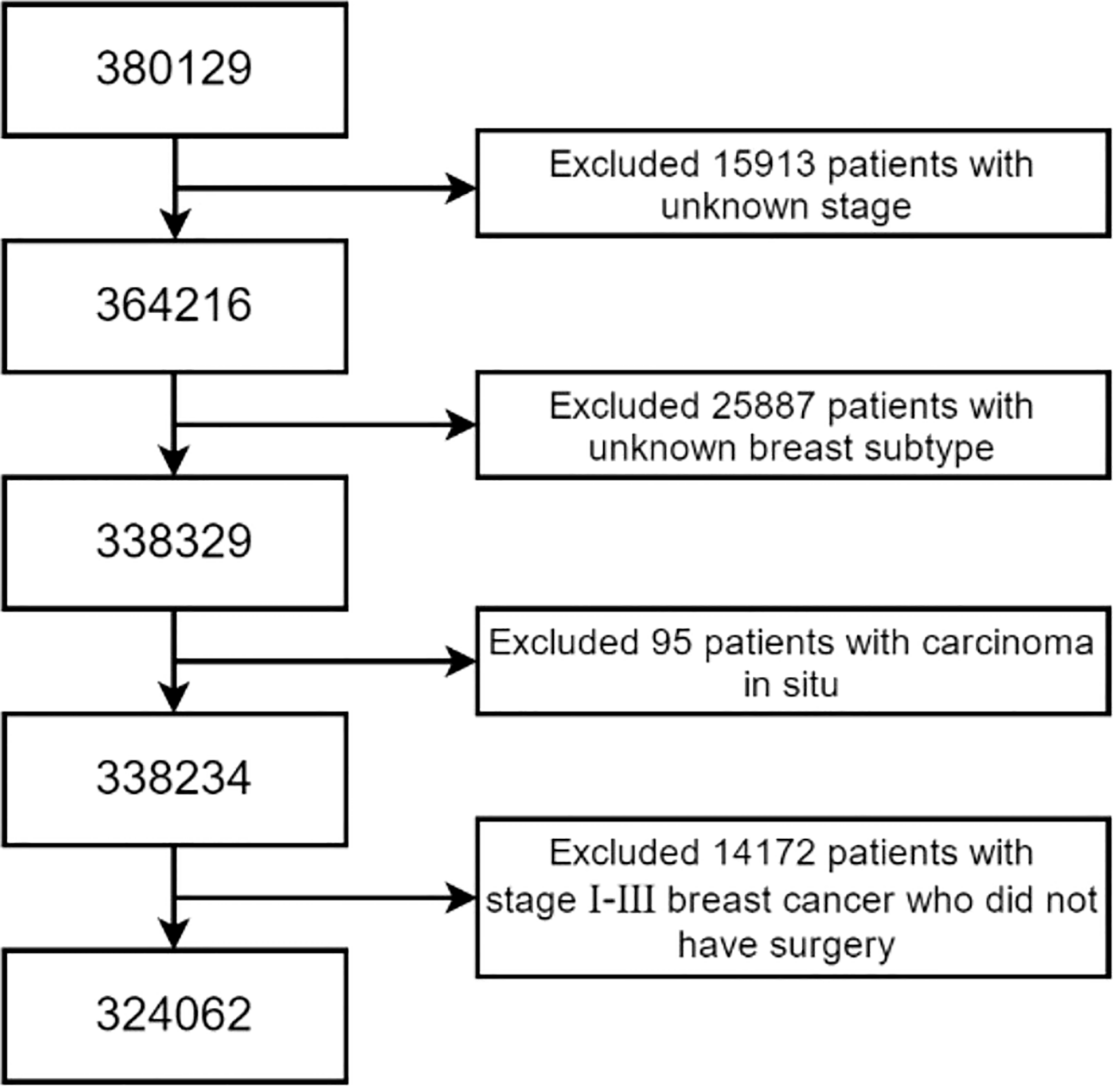
Subject selection.

We extracted the data of marital status and other clinicopathological factors of patients aged 18 years or older and with breast cancer diagnosed from January 1, 2010, to December 31, 2015, from the Surveillance, Epidemiology, and End Results (SEER)-18 database released in April 2021. The inclusion criteria were as follows: (a) the known tumor stage (AJCC 7th edition), (b) the known molecular subtype, (c) invasive cancers, and (d) Stage I–III breast cancer subjected to surgical treatment or Stage IV breast cancer. Patients who did not meet the aforementioned inclusion criteria were excluded from our study. We excluded 15,913 cases with the unknown tumor stage, 25,887 cases with the unknown molecular typing, 95 cases of carcinoma *in situ* and 14,172 cases with Stages I–III without surgical treatment (**Figure 1**). Finally, a total of 324,062 cases were included in this study.

The marital status was divided into married and unmarried (e.g., single, divorced, separated, widowed, and domestic partner). The tumor stages were divided into four groups: Stage I, Stage II, Stage III, and Stage IV. The molecular typing was divided into four groups: HR+/HER2-, HR+/HER2+, HR-/HER2-, and HR-/HER2-. The race was divided into three groups: the white, the black, and other races (including American Indian or Alaska Native and Asian or Pacific Islander). The age was divided into three groups: <35 years old group, ≥35 years and <65 years old group, and ≧65 years old group. The histological grading was divided into four groups: well differentiated (Grade I), moderately differentiated (Grade II), poorly differentiated (Grade III), and undifferentiated and anaplastic (Grade IV). The insurance status was divided into three groups: uninsured, insured, and Medicaid.

The differences in clinicopathological indexes among groups were analyzed by the Pearson *χ* ^2^ test.BCSS time was defined as the time from the diagnosis to the death due to breast cancer, and OS time was defined as the time from the diagnosis to the death due to any cause. The Kaplan–Meier survival curve was used to estimate the survival rate, while the log-rank test was used to compare the survival differences among groups. A Cox proportional hazards model was constructed for univariate and multivariate analyses and the generation of HR value and 95% CI. All statistical analyses were performed using IBM SPSS Statistics, version 22.0 (IBM Corp.). All tests were two sided, with *P <*0.05 indicating a statistically significant difference.

## Results

### Clinicopathologic Characteristics of Study Participants

A total of 324,062 cases were involved in this study, with an average age of 61.4 ± 13.3 years (median = 62.0;range:18-104 years) and a mean follow-up of 41.4 ± 21.6 months (median= 40.0 months; range: 0–83 months). The number of death events related to breast cancer was 22,274 (6.9%), and the total number of death events was 39,337 (12.1%). Further, 178,153 (55.0%), 129,549 (40.0%), and 16,360 (5.0%) of them were married, unmarried, and unknown, respectively. Patients with Stages I, II, III, and IV breast cancer accounted for 51.8%, 32.2%, 10.5%, and 5.5%, respectively. For other clinicopathological indexes, the number and proportion of patients in each subgroup are shown in **Table 1**.

**Table 1:** Clinicalpathologic characteristics of subjects according to marital status.

Characteristics	Marital status			P	Total
	Unmarried	Married	Unknown		No. (%)
	No.(%)	No.(%)	No.(%)		
All	129549 (40.0)%	178153 (55.0%)	16360 (5.0%)		324062 (100%)
Gender				<0.001	
Female	128860 (99.5%)	176525 (99.1%)	16224 (99.2%)		321609 (99.2%)
Male	689 (0.5%)	1628 (0.9%)	136 (0.8%)		2453 (0.8%)
T				<0.001	
T0/1	73540 (57.3%)	111427 (62.9%)	9955 (61.7%)		194922 (60.6%)
T2	39834 (31.0%)	50804 (28.7%)	4724 (29.3%)		95362 (29.7%)
T3	8388 (6.5%)	9729 (5.5%)	834 (5.2%)		18951 (5.9%)
T4	6536 (5.1%)	5066 (2.9%)	631 (3.9%)		12233 (3.8%)
N				<0.001	
N0	87260 (67.8%)	122699 (69.1%)	11327 (69.9%)		221286 (68.6%)
N1	29187 (22.7%)	40425 (22.8%)	3584 (22.1%)		73196 (22.7%)
N2	7412 (5.8%)	8875 (5.0%)	798 (4.9%)		17085 (5.3%)
N3	4810 (3.7%)	5516 (3.1%)	503 (3.1)		10829 (3.4%)
Stage				<0.001	
I	63358 (48.9%)	95886 (53.8%)	8608 (52.6%)		167852 (51.8%)
II	42439 (32.8%)	56761 (31.9%)	5144 (31.4%)		104344 (32.2%)
III	14691 (11.3%)	17713 (9.9%)	1593 (9.7%)		33997 (10.5%)
IV	9061 (7.0%)	7793 (4.4%)	1015 (6.2%)		17869 (5.5%)
ER				<0.001	
Negative	22100 (17.1%)	28900 (16.2%)	2714 (16.6%)		53714 (16.6%)
Positve	107437 (82.9%)	149242 (83.8%)	13643 (83.4%)		270322 (83.4%)
PR				<0.001	
Negative	36363 (28.1%)	46936 (26.4%)	4520 (27.7%)		87819 (27.2%)
Positve	92947 (71.9%)	130803 (73.6%)	11796 (72.3%)		235546 (72.8%)
HER2				<0.001	
Negative	110843 (85.6%)	151282 (84.9%)	14052 (85.9%)		276177 (85.2%)
Positve	18706 (14.4%)	26871 (15.1%)	2308 (14.1%)		47885 (14.8%)
Subtypes				<0.001	
HR+/HER2-	95701 (73.9%)	132093 (74.1%)	12192 (74.5%)		239986 (74.1%)
HR+/HER2+	13080 (10.1%)	18930 (10.6%)	1615 (9.9%)		33625 (10.4%)
HR-/HER2+	5626 (4.3%)	7941 (4.5%)	693 (4.2%)		14260 (4.4%)
HR-/HER2-	15142 (11.7%)	19189 (10.8%)	1860 (11.4%)		36191 (11.2%)
Grade				<0.001	
I	28176 (22.7%)	40987 (23.9%)	3709 (24.0%)		72872 (23.4%)
II	55191 (44.4%)	76459 (44.5%)	6995 (45.3%)		138645 (44.5%)
III	40462 (32.6%)	53773 (31.3%)	4692 (30.4%)		98927 (31.8%)
IV	393 (0.3%)	452 (0.3%)	39 (0.3%)		884 (0.3%)
Race				<0.001	
White	98503 (76.4%)	146752 (82.8%)	12621 (78.7%)		257876 (80.0%)
Black	21123 (16.4%)	12077 (6.8%)	2058 (12.8%)		35258 (10.9%)
Other races	9367 (7.3%)	18493 (10.4%)	1367 (80.5%)		29227 (9.1%)
Age group (years)				<0.001	
<35	2662 (2.1%)	3030 (1.7%)	271 (1.7%)		5963 (1.8%)
≥35,<65	61765 (47.7%)	112146 (62.9%)	8812 (53.9%)		182723 (56.4%)
≥65	65122 (50.3%)	62977 (35.3%)	7277 (44.5%)		135376 (41.8%)
Insurance				<0.001	
Uninsured	2583 (2.0%)	2091 (1.2%)	255 (1.8%)		4929 (1.5%)
Insured	103186 (80.4%)	162623 (92.1)	12443 (88.0%)		278252 (87.2%)
Medicaid	22576 (17.6%)	11931 (6.8%)	1442 (10.2%)		35949 (11.3%)
Year of diagnosis				<0.001	
2010	20115 (15.5%)	27354 (15.4%)	2231 (13.6%)		49700 (15.3%)
2011	20869 (16.1%)	28543 (16.0%)	3010 (18.4%)		52422 (16.2%)
2012	21442 (16.6%)	29412 (16.5%)	2955 (18.1%)		53809 (16.6%)
2013	22001 (17.0%)	30331 (17.0%)	2681 (16.4%)		55013 (17.0%)
2014	22222 (17.2%)	30728 (17.2%)	2847 (17.4%)		55797 (17.2%)
2015	22900 (17.7%)	31785 (17.8%)	2636 (16.1%)		57321 (17.7%)

### Survival Analysis of All Participants According to Marital Status

**Figure 2:**
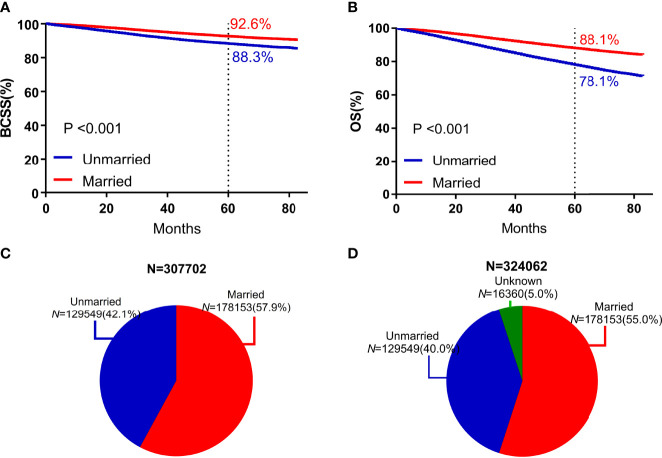
Survival curves and subject proportions according to marital statuses. BCSS **(A)** and OS **(B)** according to marital statuses were depicted. Subject proportions according to marital statuses without the unknown marital status **(C)** and with the unknown marital status **(D)** were also depicted.

Compared with unmarried patients, married patients exhibited significantly higher 5-year BCSS (92.6% vs 88.3%, *P* < 0.001) and 5-year OS (88.1% vs 78.1%, *P* < 0.001) (**Figure 2**).

Based on the univariate analysis, marital status, sex, T stage, N stage, ER status, PR status, HER2 status, molecular typing, historical grading, age, insurance status, and year of diagnosis were associated with BCSS and OS. Compared with the unmarried subgroup, the HR of BCSS of the married subgroup was 0.583 (95% CI = 0.67–0.599, *P* < 0.001), and the HR of OS was 0.498 (95% CI = 0.488–0.509, *P* < 0.001). According to the multivariate analysis, after adjusted for sex, T stage, N stage, ER status, PR status, HER2 status, histological grading, age, insurance status, and year of diagnosis, the marital status was still an independent predictor of BCSS and OS. Compared with the unmarried subgroup, the HR of BCSS of the married subgroup was 0.775 (95% CI = 0.753–0.797, *P* < 0.001), and the HR of OS was 0.667 (95% CI = 0.653–0.681, *P* < 0.001) (**Table 2**).

**Table 2:** Univariable and multivariable analyses regarding BCSS and OS.

Characteristics	BCSS						OS						
	Univariable analysis			Multivariable analysis			Univariable analysis			Multivariable analysis		
	HR	95%CI	P	HR	95%CI	P	HR	95%CI	P	HR	95%CI	P	
Marital status			<0.001			<0.001			<0.001			<0.001	
Unmarried	ref			ref			ref			ref			
Married	0.583	0.567-0.599	<0.001	0.775	0.753-0.797	<0.001	0.498	0.488-0.509	<0.001	0.667	0.653-0.681	<0.001	
Unknown	0.813	0.766-863	<0.001	0.919	0.865-0.977	0.007	0.78	0.746-0.815	<0.001	0.875	0.837-0.916	<0.001	
Gender			<0.001			<0.001			<0.001			<0.001	
Female	ref			ref			ref			ref			
Male	1.479	1.301-1.681	<0.001	1.247	1.096-1.419	0.001	1.896	1.740-2.065	<0.001	1.54	1.413-1.679	<0.001	
T			<0.001			<0.001			<0.001			<0.001	
T0/1	ref			ref			ref			ref			
T2	3.976	3.833-4.123	<0.001	2.392	2.301-2.485	<0.001	2.265	2.212-2.319	<0.001	1.775	1.731-1.821	<0.001	
T3	9.073	8.679-9.485	<0.001	4.347	4.143-4.561	<0.001	4.034	3.900-4.172	<0.001	2.822	2.721-2.927	<0.001	
T4	24.082	23.115-25.089	<0.001	8.389	8.003-8.793	<0.001	10.287	9.976-10.609	<0.001	5.305	5.119-5.498	<0.001	
N			<0.001			<0.001			<0.001			<0.001	
N0	ref			ref			ref			ref			
N1	3.582	3.469-3.699	<0.001	2.094	2.024-2.168	<0.001	1.942	1.897-1.987	<0.001	1.443	1.408-1.480	<0.001	
N2	6.068	5.817-6.330	<0.001	2.558	2.444-2.677	<0.001	3.01	2.909-3.114	<0.001	1.718	1.657-1.782	<0.001	
N3	11.048	10.598-11.517	<0.001	3.67	3.505-3.842	<0.001	5.141	4.968-5.320	<0.001	2.419	2.329-2.512	<0.001	
Stage			<0.001						<0.001				
I	ref						ref						
II	3.899	3.711-4.097	<0.001				1.866	1.816-1.918	<0.001				
III	13.158	12.528-13.820	<0.001				4.125	4.004-4.250	<0.001				
IV	67.848	64.776-71.066	<0.001				18.371	17.873-18.884	<0.001				
ER			<0.001			<0.001			<0.001			<0.001	
Negative	ref			ref			ref			ref			
Positve	0.328	0.319-0.337	<0.001	0.726	0.699-0.755	<0.001	0.474	0.463-0.484	<0.001	0.761	0.738-0.786	<0.001	
PR			<0.001			<0.001			<0.001			<0.001	
Negative	ref			ref			ref			ref			
Positve	0.34	0.331-0.349	<0.001	0.609	0.587-0.632	<0.001	0.491	0.481-0.500	<0.001	0.712	0.692-0.732	<0.001	
HER2			<0.001			<0.001			<0.001			<0.001	
Negative	ref			ref			ref			ref			
Positve	1.342	1.298-1.389	<0.001	0.696	0.672-0.721	<0.001	1.109	1.079-1.140	<0.001	0.758	0.737-0.779	<0.001	
Subtype			<0.001						<0.001				
HR+/HER2-	ref						ref						
HR+/HER2+	1.468	1.405-1.533	<0.001				1.15	1.111-1.189	<0.001				
HR-/HER2+	2.415	2.294-2.542	<0.001				1.629	1.561-1.700	<0.001				
HR-/HER2-	3.432	3.327-3.540	<0.001				2.326	2.269-2.385	<0.001				
Grade			<0.001			<0.001			<0.001			<0.001	
I	ref			ref			ref			ref			
II	2.852	2.684-3.031	<0.001	1.763	1.658-1.875	<0.001	1.517	1.469-1.567	<0.001	1.157	1.119-1.196	<0.001	
III	7.578	7.147-8.035	<0.001	2.747	2.581-2.923	<0.001	2.748	2.662-2.837	<0.001	1.531	1.477-1.586	<0.001	
IV	10.812	9.202-12.702	<0.001	3.052	2.593-3.592	<0.001	3.791	3.326-4.319	<0.001	1.764	1.546-2.012	<0.001	
Race			<0.001			<0.001			<0.001			<0.001	
White	ref			ref			ref			ref			
Black	1.897	1.833-1.963	<0.001	1.197	1.156-1.240	<0.001	1.548	1.506-1.591	<0.001	1.138	1.106-1.170	<0.001	
Other races	0.804	0.763-0.848	<0.001	0.786	0.745-0.829	<0.001	0.697	0.668-0.726	<0.001	0.742	0.711-0.773	<0.001	
Age group (years)			<0.001			<0.001			<0.001			<0.001	
<35	ref			ref			ref			ref			
≥35,<65	0.604	0.557-0.655	<0.001	1.038	0.957-1.126	0.372	0.691	0.641-0.745	<0.001	1.056	0.979-1.139	0.161	
≥65	0.727	0.670-0.789	<0.001	1.683	1.550-1.828	<0.001	1.489	1.382-1.605	<0.001	2.715	2.517-2.929	<0.001	
Insurance			<0.001			<0.001			<0.001			<0.001	
Uninsured	ref			ref			ref			ref			
Insured	0.391	0.362-0.421	<0.001	0.645	0.597-0.697	<0.001	0.56	0.524-0.599	<0.001	0.631	0.589-0.675	<0.001	
Medicaid	0.842	0.778-0.913	<0.001	0.87	0.803-0.943	0.001	1.032	0.962-1.107	0.374	0.916	0.854-0.983	0.015	
Year of diagnosis			0.005			0.625			<0.001			0.313	
2010	ref			ref			ref			ref			
2011	1.004	0.965-1.044	0.857	1.022	0.983-1.063	0.272	0.994	0.966-1.024	0.706	1.002	0.973-1.032	0.885	
2012	0.965	0.926-1.006	0.094	0.994	0.953-1.036	0.77	0.985	0.955-1.017	0.355	0.996	0.965-1.027	0.791	
2013	0.971	0.929-1.015	0.191	1.025	0.980-1.071	0.286	0.965	0.933-0.998	0.04	0.988	0.954-1.022	0.469	
2014	0.943	0.897-0.991	0.021	1.008	0.959-1.060	0.757	0.955	0.919-0.992	0.019	0.988	0.950-1.027	0.529	
2015	0.903	0.850-0.960	0.001	0.991	0.932-1.054	0.778	0.899	0.857-0.943	<0.001	0.947	0.902-0.993	0.026	

### Survival Analysis for Subgroups of Sex, Stage, Subtype, and Age

**Figure 3:**
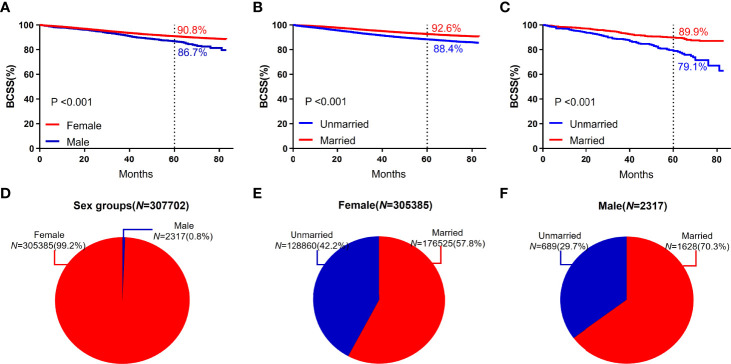
BCSS curves according to the sex groups. **(A)** and subject proportions of the sex groups **(D)**. Survival curves of each marital status for female **(B)** and male **(C)** and subject proportions of each marital status for female **(E)** and male **(F)** were depicted.

The study included 2317 male patients, accounting for only 0.8%. The 5-year BCSS (86.7%) of male participants was lower than that of female participants (90.8%). The five-year BCSS of married and unmarried male and female patients was (89.9% vs 79.1%, *P* < 0.001) and (92.6% vs 88.4%, *P* < 0.001), respectively (**Figure 3**).

**Figure 4:**
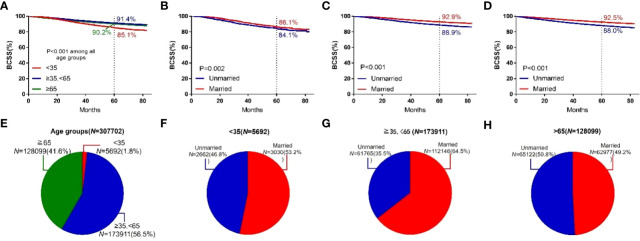
BCSS curves according to the age groups. **(A)** and subject proportions of the age groups **(E)**. Survival curves of each marital status for <35 years old **(B)**, ≥35, <65years old **(C)** and ≧65years old **(D)** and subject proportions of each marital status for <35 years old **(F)**, ≥35, <65years old **(G)** and ≧65years old **(H)** were depicted.

The 5-year BCSS in subgroups with ages between 35 and 65 years (91.4%) was higher than that in the subgroups with age ≥65 years (90.2%) and <35 years (85.1%). In each age group, the 5-year BCSS of married patients was better than that of unmarried patients (**Figure 4**). Patients with low histological grading had a higher 5-year BCSS. For different histological grading, married patients had a higher 5-year BCSS (**Figure 4**).

**Figure 5:**
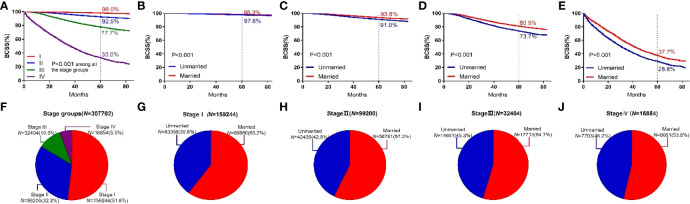
BCSS curves according to the stage groups. **(A)** and subject proportions of the stage groups **(F)**. Survival curves of each marital status for stage I **(B)**, stage II **(C)**, stage III **(D)** and stage IV **(E)** and subject proportions of each marital status for stage I **(G)**, stage II **(H)**, stage III **(I)** and stage IV **(J)** were depicted.

In this study, breast cancer was divided into Stages I, II, III, and IV, and the 5-year BCSS was 98.0%, 92.5%, 77.7%, and 33.0%, respectively. Significant differences were observed among groups (*P* < 0.001). For different subgroups, the 5-year BCSS of married and unmarried patients was (98.3% vs 97.6%, *P* < 0.001), (93.8% vs 91.0%, *P* < 0.001), (80.9% vs 73.7%, *P* < 0.001), and (37.7% vs 28.8%, *P* < 0.001), respectively, as shown in **Figure 5**.

**Figure 6:**
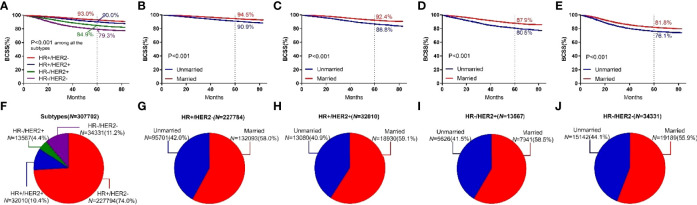
BCSS curves according to the subtypes. **(A)** and subject proportions of the subtypes **(F)**. Survival curves of each marital status for HR+/HER2- **(B)**, HR+/HER2+ **(C)**, HR-/HER2+ **(D)** and HR-/HER2- **(E)** and subject proportions of each marital status for HR+/HER2- **(G)**, HR+/HER2+ **(H)**, HR-/HER2+ **(I)** and HR-/HER2- **(J)** were depicted.

Among the four breast cancer subtypes, HR+/HER2− subtype showed the highest 5-year BCSS rate (93.0%).HR+/HER2+ subtype showed a higher 5-year BCSS rate (90.0%) than HR−/HER2+ (84.9%) and HR−/HER2− subtypes (79.3%). HR−/HER2− subtype showed the lowest 5-year BCSS rate. Among all four types of breast cancer, the 5-year BCSS of married patients and unmarried patients was (94.5% vs 90.9%, *P <*0.001), (92.4% vs 86.8%, *P* < 0.001), (87.9% vs 80.5%, *P* < 0.001), and (81.8% vs 76.1%, *P* < 0.001), as shown in **Figure 6**.

### Multivariable and Interaction Analyses of Subgroups Corresponding to Different Clinical-Pathological Factors

**Figure 7:**
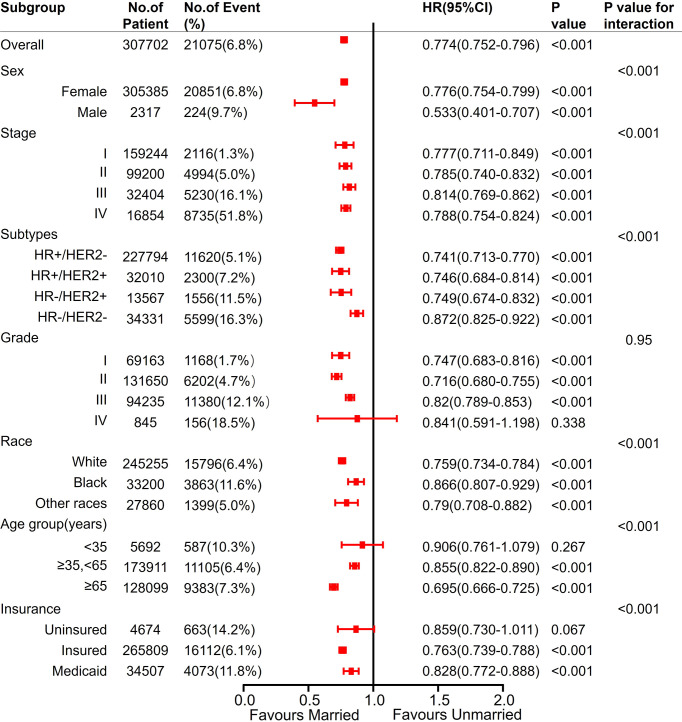
Analyses of BCSS (breast cancer-specific survival) for married vs unmarried patients in each subgroup after adjusted by other clinicalpathological factors. Hazard Ratio (HR) estimates for BCSS are indicated by rectangles, and 95% confidence intervals (95% CI) are indicated by the crossing horizontal lines.

In each subgroup of sex, stage, molecular typing, histological grading, age, and insurance status, Cox regression analysis was conducted with BCSS and OS as the observation endpoints. The other five variables and marital status were involved in the Cox model. Being married was an independent predictor of better BCSS, except for Grade IV, age <35 years, and uninsured subgroups. Except for histological grading, all the other variables had interaction effects with marital status (**Figure 7**).

**Figure 8:**
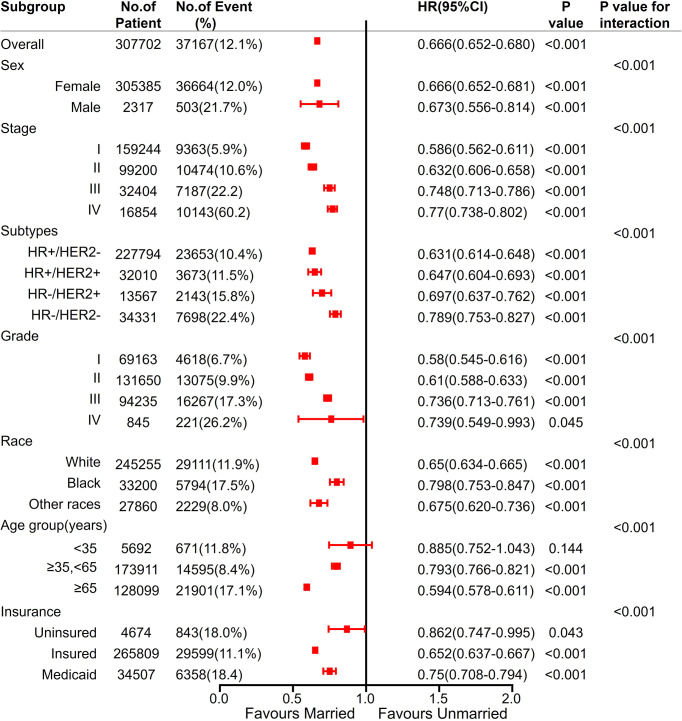
Analyses of OS (Overall survival) for married vs unmarried patients in each subgroup after adjusted by other clinicalpathological factors. Hazard Ratio (HR) estimates for BCSS are indicated by rectangles, and 95% confidence intervals (95% CI) are indicated by the crossing horizontal lines.

Moreover, being married was an independent predictor of better OS, except for age <35 years subgroup. All the variables had interaction effects with marital status (**Figure 8**).

## Discussion

This study investigated the relationship between marital status and BCSS and OS in 324,062 patients with invasive breast cancer in the SEER database. First, by analyzing the relationship between marital status and survival in each subpopulation, we found that marital status was an independent prognostic factor among different stages of breast cancer. Therefore, the effect of marital status on prognosis could not be explained simply by the stage at the time of diagnosis. Then, our results indicated that the effect of marital status on survival might be different in different subgroups with breast cancer. Especially, the marital status of patients with breast cancer aged <35 had no significant effect on survival. Additionally, we also found that for different sex, molecular typing, and insurance status, marital status was an independent prognostic factor for breast cancer.

Aizer et al. (4) analyzed the survival rates of patients diagnosed as the top 10 tumors with the highest tumor-related mortality in the SEER database from 2004 to 2008. They found that marital status could affect the tumor stage, treatment, and tumor-related death at the time of diagnosis. Compared with unmarried patients with breast cancer, married patients had fewer advanced lesions at the time of diagnosis [odds ratio (OR) = 0.60, 95% CI = 0.58–0.63], higher proportion of receiving treatment (OR = 1.54, 95% CI = 1.42–1.66), and lower breast cancer–related mortality (HR = 0.78, 95% CI = 0.74–0.81).

Chen et al. (8) performed mediation analyses to investigate the intermediate factors of marital status affecting the survival of patients with cancer, demonstrating that marital status could affect the survival of patients by affecting the stage of breast cancer at the time of diagnosis. This study found that the proportion of married patients with the advanced stage at the time of diagnosis was lower than that of unmarried patients (4.4% vs 7.0%).

For patients with different stages of breast cancer, the relationship between marital status and survival was analyzed; the survival of married patients was better than that of unmarried patients in each stage. Consequently, we speculated that the marital status affected the survival of patients with breast cancer not just by influencing the stage at the time of diagnosis. Patients with different marital statuses might have different mental states and living habits. These factors might also affect the ability of patients with breast cancer to reintegrate into society, besides impacting the emotional recovery and even postoperative recovery (17–19).

Previous studies demonstrated that marital status was closely related to survival for aged patients with breast cancer (20). This study was the first to report that the relationship between marital status and survival was not consistent in each age subgroup. For patients with breast cancer aged <35 years, the prognosis of married and unmarried patients showed no differences. This was not consistent with previous studies (21). The analysis showed that the different correction factors added in the two studies contributed to the difference in the final results. Stage and molecular typing were involved in our multivariate analysis, both of which were closely related to the prognosis of breast cancer. Previous studies showed that compared with patients with breast cancer aged <35 years had an advanced stage, and the proportion of HER2-positive and triple-negative breast cancer was higher (22). Patients with early breast cancer aged <35 years had worse 5- and 10-year survival (23, 24), and marital status was irrelevant to this conclusion. Adekolujo at al. (25) analyzed the marital status and survival of 3761 patients diagnosed with breast cancer in the SEER database from 1990 to 2011, and found that unmarried patients had an advanced stage and worse diagnosis. In this study, the marital status might have had a more significant impact on the survival of male patients with breast cancer than on female patients (BCSS, HR: 0.533 vs. 0.776). Compared with uninsured and Medicaid patients with breast cancer, insured patients with breast cancer had an earlier stage and better prognosis (26, 27). Molecular typing (28), race (29, 30), and histological grading (31) were also independent factors for the prognosis of breast cancer. In this study, we found that the influence of marital status on prognosis was independent of these factors.

This study also had limitations. First, this was a retrospective study, which had inherent defects, including data bias. Then, little information was available about breast cancer treatment in the SEER database, and hence it was impossible to know the impact of marital status on treatment selection and compliance. Breast cancer does not contribute to the marital breakdown (32). However, a disharmonious partnership in marriage could delay the postoperative recovery of breast cancer and cause a worse prognosis (33). Hence, the prognosis of even married patients varied due to different partnerships. Emotional support was beneficial to the survival of unmarried patients (34). However, this study could not answer how additional psychosocial and emotional supports might improve the survival of unmarried patients and the degree of improvement. Nevertheless, this study might be valuable for understanding the relationship between marital status and prognosis. Due to the large sample size, we could analyze the subgroup of each clinicopathological index by the univariate and multivariate analysis. The final conclusion might help understand how different clinicopathological indexes affect the relationship between marital status and prognosis, and assist the unmarried population who really need emotional intervention.

## Conclusions

This study found for the first time that among patients with different stages of breast cancer, married ones had a better prognosis. Therefore, the effect of marital status on prognosis could not be explained simply by the stage of breast cancer at the time of diagnosis. We also found for the first time that in the subgroup of patients aged <35 years, marital status was not associated with the prognosis of breast cancer. Further in-depth research is needed to clarify this phenomenon. Additionally, it was reported for the first time that marital status was an independent prognosis factor for breast cancer regardless of the molecular typing. These conclusions provide a basis for us to further understand the relationship between marital status and prognosis of breast cancer, and also highlight the necessity of providing emotional support to unmarried patients with breast cancer.

## Data Availability Statement

The original contributions presented in the study are included in the article/supplementary material. Further inquiries can be directed to the corresponding author.

## Ethics Statement

Ethical approval was not provided for this study on human participants because the data accrued was anonymized at source by the SEER-18 database, therefore no consent was deemed necessary. Written informed consent for participation was not required for this study in accordance with the national legislation and the institutional requirements.

## Author Contributions

DJ participated in the study design and writing the draft manuscript. YM participated in data collection, collation, and statistical analysis. JZ and HD contributed to study design, results interpretation, and manuscript editing. YY, YZ, and XG participated in the analysis of the results and the revision of the manuscript. ZL conceived the study, interpreted data, and edited the manuscript. All authors contributed to the article and approved the submitted version.

## Conflict of Interest

The authors declare that the research was conducted in the absence of any commercial or financial relationships that could be construed as a potential conflict of interest.

## Publisher’s Note

All claims expressed in this article are solely those of the authors and do not necessarily represent those of their affiliated organizations, or those of the publisher, the editors and the reviewers. Any product that may be evaluated in this article, or claim that may be made by its manufacturer, is not guaranteed or endorsed by the publisher.

## Abbreviations

SEER, Surveillance, epidemiology, and end results; BCSS, Breast cancer–specific survival; OS, Overall survival; HR, Hazard ratio; HR+, Hormone receptor positive; HR-, Hormone receptor negative; HER2, Human epidermal growth factor receptor-2.
